# Nonproteolytic ubiquitination regulates chromatin occupancy by the NCoR/SMRT/HDAC3 corepressor complex in MCF-7 breast cancer cells

**DOI:** 10.1073/pnas.2502805122

**Published:** 2025-04-30

**Authors:** Giulio Ferrero, Maria Dafne Cardamone, Francesca Luca, Eliot Bourk, Laura Ricci, Wen Liu, Yuan Gao, Giulia Burrone, Akhirah Muhammad, Stefanie Chan, Emma Smith, Ting-Yu Claire Fan, Santina Cutrupi, Ivan Garcia-Bassets, Michele De Bortoli, Michael G. Rosenfeld, Valentina Perissi

**Affiliations:** ^a^Department of Clinical and Biological Science, University of Torino, Orbassano (Torino) 10043, Italy; ^b^Department of Biochemistry and Cell Biology, Chobanian and Avedisian School of Medicine, Boston University, Boston, MA 02118; ^c^Department of Medicine, School of Medicine, University of California San Diego, La Jolla, CA 92037; ^d^Department of Computer Science, University of Torino, Torino 10149, Italy

**Keywords:** transcription, corepressor, ubiquitin, breast cancer

## Abstract

Tight regulation of gene expression is achieved through the coordinated action of transcription factors and cofactors that often can act as both repressors and activators in response to regulatory signals, with their activity modulated by context-specific signal transduction pathways that also impinge on their transient and cyclical recruitment to chromatin. However, the mechanisms underlying the intricate interplay between the regulatory strategies controlling cofactors’ activity and localization across subcellar domains remain poorly understood. Here, we investigated the role of G-Protein Pathway Suppressor 2 (GPS2), a transcriptional cofactor critical for maintaining cellular homeostasis via regulation of mitochondrial biogenesis, stress response, lipid metabolism, insulin signaling, and inflammation, in MCF-7 breast cancer cells. By integration of biochemical assays with genome-wide RNA sequencing and Chromatin immunoprecipitation-Seq analyses, we show that nuclear GPS2 is required for licensing histone deacetylase 3 recruitment to chromatin via restricted ubiquitination by tumor necrosis factor receptor-associated factor 6 (TRAF6), an E3 ubiquitin ligase previously shown to regulate the switch from repressive to activating functions of the nuclear receptor corepressor (NCoR)/silencing mediator of retinoic acid and thyroid hormone receptor (SMRT) complex and here unexpectedly found to translocate to the nucleus in response to IL-1β stimulation. Nuclear TRAF6 is recruited to chromatin via direct interaction with the corepressors NCoR/SMRT, and TRAF6-mediated ubiquitination of TGF-beta activated kinase 1 (MAP3K7) binding protein 2 (TAB2), a facultative component of the NCoR/SMRT complex, contributes to corepressor clearance from target regulatory regions. Together, these results reveal an exquisite mechanism for coordinating the local regulation of cofactor activity with proinflammatory signaling pathways.

## Significance

Cells tightly control gene expression through the combined actions of many transcription factors and cofactors responding to developmental and environmental signals. This study uncovers an unexpected mechanism by which inflammatory signals influence gene expression in breast cancer cells by regulating the transcriptional activity of the nuclear receptor corepressor (NCoR)/silencing mediator of retinoic acid and thyroid hormone receptor (SMRT)/histone deacetylase 3 (HDAC3) corepressor complex through tumor necrosis factor receptor-associated factor 6 (TRAF6)-dependent ubiquitination events. These findings reveal an intricate interplay between the mechanisms regulating the function and clearance of chromatin remodeling complexes by immune signaling pathways across subcellular compartments.

Gene expression modulation is achieved through the complex interplay of repression and activation events mediated by many transcriptional regulators, including several exhibiting dual functions as both corepressors and coactivators (1). This is exemplified by the nuclear receptor corepressor (NCoR)/silencing mediator of retinoic acid and thyroid hormone receptor (SMRT) complex, initially identified and characterized as a corepressor complex carrying histone deacetylation activity and later shown to associate with gene activation as well (2–7). As the switch between these opposite functions has been associated with phosphorylation events promoting the deacetylation of distinct targets (5–11), recruitment and regulation of histone deacetylase 3 (HDAC3)—the main chromatin remodeling enzyme in the NCoR/SMRT complex—are key determinants of the transcriptional outcome. Interplay between the various components of the NCoR/SMRT complex plays a central role in this context, with HDAC3 enzymatic activity being directly dependent on its interaction with a deacetylase-activating domain in NCoR/SMRT (2, 11–15).

In addition to HDAC3, integral components of the NCoR/SMRT corepressor complex include the histone binding and exchange factors TBL1/TBLR1 and the regulatory subunit G-Protein Pathway Suppressor 2 (GPS2) (3, 13, 16–19). GPS2 is a small, conserved subunit initially isolated during a screening for suppressors of Ras activation in yeast and later shown to regulate multiple processes critical to maintaining cellular homeostasis in mammalian cells, including lipid metabolism, insulin signaling, mitochondrial biogenesis and bioenergetics, and inflammation (19–32). These complementary functions are mediated by a combination of genomic and nongenomic mechanisms that reflects GPS2 dynamic localization to various subcellular locations (22, 23, 25–29, 33, 34). Nonetheless, the molecular basis of GPS2 activity appears consistent across different processes and locations as GPS2-mediated inhibition of non-proteolytic-ubiquitination activity is important for both transcriptional regulation of PPARγ target genes and nuclear-encoded mitochondrial genes (neMITO) in the nucleus, translational control of antioxidant transcripts on the outer mitochondrial membrane (35) and posttranslational regulation of Phosphatidylinositol 3-kinase (PI3K)/AKT signaling and Tumor Necrosis Factor-alpha/Toll-like receptor 4 (TLR4) proinflammatory pathways in the cytosol (25–29).

Ubiquitination is a reversible modification that is achieved via the sequential activity of several classes of enzymes, including a ubiquitin (Ub)-activating enzyme (E1), an Ub-conjugating enzyme (E2), and an Ub ligase (E3) (36, 37). Polyubiquitination of target proteins with chains of different topologies can promote protein degradation or, as in the case of other posttranslational modifications, influence protein functions and interactions (36–40) GPS2 inhibits the activity of Ubc13/Ube2N, an E2 ubiquitin-conjugating enzyme responsible for the synthesis of nonproteolytic K63 ubiquitin chains, and previous studies have linked GPS2-mediated inhibition of Ubc13 activity to both nuclear and extranuclear events (25–29). In differentiating adipocytes, GPS2-mediated inhibition of Ubc13 activity regulates gene expression via the stabilization of a histone demethylase, KDM4A/JMJD2A, responsible for removing the repressive H3K9me3 marks from selected PPARγ target genes (25). Similarly, under conditions of mitochondrial stress, GPS2-mediated inhibition of Ubc13 activity promotes chromatin remodeling and activation of stress response and neMITO by promoting H3K9 demethylation (29). While in these contexts GPS2 promotes gene activation, its role in mediating the repression of proinflammatory genes and estrogen receptor targets as part of the NCoR/SMRT complex is also well established, as supported by the transcriptomic analysis of various Knock-out (KO)/Knock-down models (19, 21, 23, 24, 32, 34). However, it remains to be determined whether the inhibition of Ubc13 activity similarly contributes to GPS2 repressive functions.

Recent studies have begun uncovering an unexpected role for K63 ubiquitination in the modulation of HDAC3 activity. For instance, HDAC3 ubiquitination by the E3 ligase TRAF6 leads to *MYC* upregulation in hepatocellular carcinoma due to dissociation of K63-ubiquitinated HDAC3 from the MYC promoter (41). Similarly, the downregulation of USP38, a K63 deubiquitinase, in colorectal cancer is associated with increased ubiquitination of HDAC3 and upregulation of cancer stem cell–related genes (42). Still, the role of K63 ubiquitination in the modulation of HDAC3 activity has not been fully elucidated. Here, we interrogated whether GPS2 inhibitory role against K63 ubiquitination is relevant for the regulation of HDAC3-regulated genes. Our results reveal that lack of GPS2 leads to aberrant HDAC3 ubiquitination and dismissal of HDAC3 from most target genes, including both HDAC3-activated and -repressed genes. Our studies also uncover the molecular basis of TRAF6-mediated regulation of the NCoR/SMRT complex activity, including identifying and characterizing another component of the complex, TGF-beta activated kinase 1 (MAP3K7) binding protein 2, as a target of ubiquitination-dependent regulation.

## Results

### GPS2 Represses c-Myc Gene Expression through Inhibition of HDAC3 Ubiquitination.

**Fig. 1.**
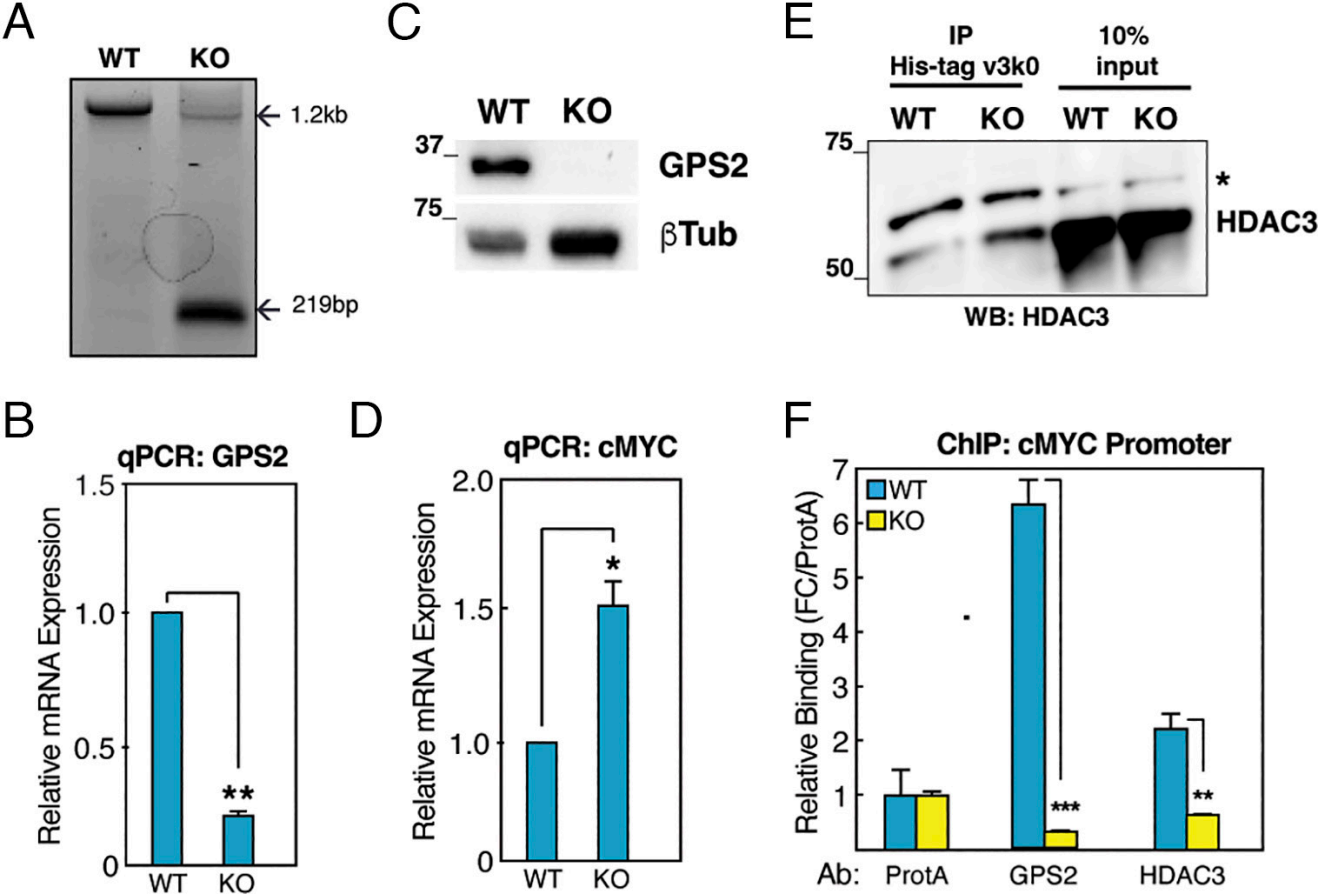
(*A*) GPS2 deletion by CRISPR/Cas9 genome editing is validated by genomic PCR. PCR fragment of 219 bp is indicative of deletion of exons 2-6. (*B* and *C*) RT-qPCR (*B*) and western blot analysis (*C*) of GPS2 levels in GPS2 WT and KO MCF-7 cells. RTqPCR data are normalized to Human Cyclophilin A. *P < 0.05 vs. control, Student’s *t* test. Data are represented as mean ± SEM. (*D*) Expression of c-myc measured by RTqPCR with data normalized to human Cyclophilin A. *P < 0.05 vs. control, Student’s *t* test. Data are represented as mean ± SEM. (*E*) Ubiquitination of HDAC3. Immunoprecipitation analysis was performed on nuclear extracts using a K63-ubiquitin-binding column (His-tag Vx3k0) as described in ref. 35. Immunoprecipitated fraction was analyzed by immunoblotting with anti-HDAC3 antibody. * indicates an aspecific band. (*F*) Chromatin immunoprecipitation (ChIP) for HDAC3 and GPS2 on the *MYC* gene promoter. Bar graphs represent the sample mean of three technical replicates. *P value < 0.05; **P value < 0.01; ***P value < 0.001, Student’s *t* test.

We previously reported that GPS2 deletion in triple-negative MDA-MB231 breast cancer cells is associated with the upregulation of *MYC* and MYC-dependent target genes (43). However, the mechanism underlying GPS2-mediated regulation of c-Myc expression remained to be determined. To address this question and dissect the role of GPS2, and other components of the NCoR/SMRT corepressor complex, in the transcriptional regulation of *MYC* expression, we switched to Estrogen Receptor α-positive MCF-7 cells, which have lower basal expression of c-Myc than MDA-MB231 cells (44). GPS2 deletion in MCF-7 cells was performed by CRISPR/Cas9 genome editing (Fig. 1*A*), as previously done for MDA-MB231 cells (43). Individual clones were validated by sequencing, and it was confirmed that GPS2-KO cells exhibited the expected decrease in GPS2 RNA and protein levels compared to the parental cells (Fig. 1 *B* and *C*). Comparison of GPS2 WT and KO MCF-7 cells by RTqPCR also confirmed the upregulation of *MYC* expression in the absence of GPS2 in this model (Fig. 1*D*).

Previous studies indicate that GPS2 modulates transcriptional activation by regulating chromatin remodeling through stabilization of a histone methyltransferase [Cardamone et al. (25), 2018]. We speculated that a similar strategy might be employed for modulating gene repression, with GPS2’s role within the NCoR/SMRT complex being critical for licensing the availability of a different chromatin remodeling enzyme. Based on previous studies showing aberrant TRAF6/Ubc13 activity in the absence of GPS2 (26, 27), and TRAF6-mediated ubiquitination of HDAC3 promoting derepression of the *MYC* gene (41), we hypothesized that GPS2 regulates HDAC3 availability and HDAC3-dependent repression of *MYC* via inhibition of TRAF6 activity. To begin investigating this hypothesis, we asked whether GPS2 maintains *MYC* under negative regulation through modulation of HDAC3 ubiquitination. First, to monitor the impact of GPS2 deletion on HDAC3 ubiquitination, we performed immunoprecipitation of nuclear proteins using a K63-specific ubiquitin binding (UBD) column (His-tag Vx3k0) (35), followed by western blotting for HDAC3. The amount of HDAC3 purified by the K63-UBD was higher in GPS2-KO cells compared to WT (Fig. 1*E*), in accord with previous results showing elevated TRAF6 activity in the absence of GPS2 (26, 35). To investigate the impact of the increase in HDAC3 ubiquitination on c-Myc transcriptional regulation, we measured GPS2 and HDAC3 recruitment to the *MYC* promoter by chromatin immunoprecipitation (ChIP). As expected, both cofactors were bound to the promoter in WT MCF-7 cells (Fig. 1*F*), whereas HDAC3 recruitment to DNA was impaired in the absence of GPS2 (Fig. 1*F*). Together, these results confirm that GPS2 deletion promotes c-Myc upregulation through unrestricted ubiquitination and dismissal of HDAC3 from the *MYC* promoter.

### GPS2 KO Induces a Large-Scale Dismissal of HDAC3 Chromatin Binding.

Next, we asked whether this regulatory strategy was specific to MYC or extended to other genes. To address this question, we explored the effect of GPS2 silencing on the recruitment of HDAC3 to chromatin at the whole genome level. Chromatin Immuno-Precipitation–Sequencing (ChIP-Seq) for GPS2 and HDAC3 in WT and GPS2-KO MCF-7 cells was performed as before (Dataset S1*A*) (29, 45). The analysis of GPS2 peaks in WT cells highlighted 4,875 significant ChIP-Seq peaks, of which 96.74% were associated with the expected decrease in GPS2 occupancy in KO cells (Dataset S1*B*), thus confirming the specificity of the detected signal. Most of the GPS2 peaks were localized at gene introns (49.83%) or intergenic regions (32.98%), while 405 peaks (8.31%) were detected at gene promoters (Dataset S1*C*). Genes associated with a GPS2 peak were functionally enriched in heterogeneous processes, including those related to cell migration [e.g., *regulation of epithelial cell migration (GO:0010632), regulation of focal adhesion disassembly (GO:0120182)*] and metabolism [e.g., *cellular glucose homeostasis (GO:0001678) and proteoglycan metabolic process (GO:0006029)*] (Dataset S1*D*).

**Fig. 2.**
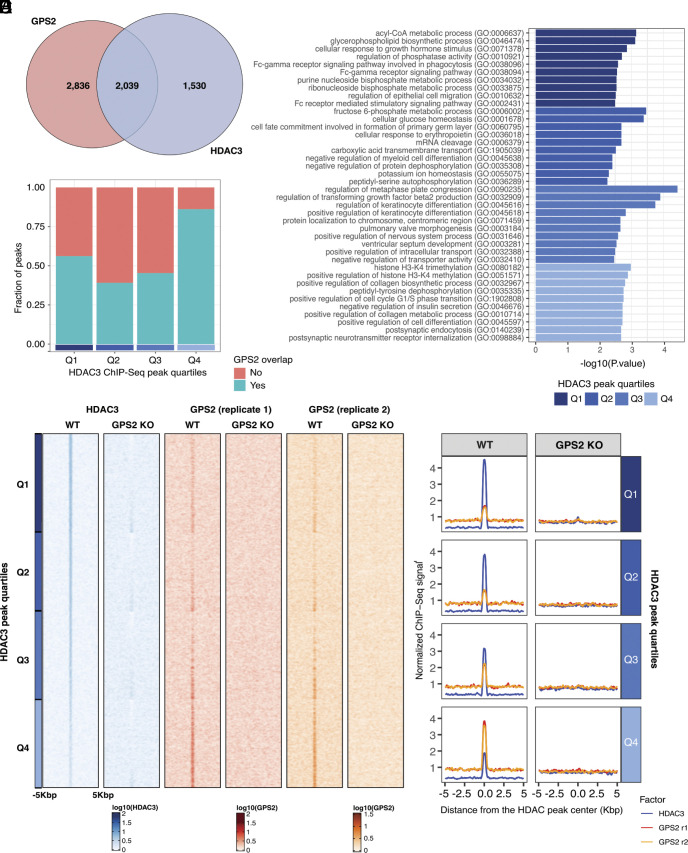
(*A*) Venn diagram showing the number of peaks overlapped between the HDAC3 and GPS2 ChIP-Seq experiments. (*B*) Bar plot reporting the fraction of HDAC3 peaks in each peak quartile overlapping a GPS2 chromatin binding in WT cells. (*C*) The bar plot shows the top 10 significant biological processes enriched in genes annotated within 10 kbp from HDAC3 peaks in each peak quartile. (*D*) Peak density heatmap reporting the normalized HDAC3 and GPS2 ChIP-Seq signals measured in a ±5 kbp window centered on the significant HDAC3 peaks obtained in the control condition. The peaks are first sorted based on the significance of HDAC3 ChIP enrichment with respect to the input samples and then sorted based on the significance of the decrease in HDAC3 binding within each quartile. The signals measured in GPS2-KO cells and the HDAC3 peak quartiles are also reported. (*E*) Average normalized HDAC3 and GPS2 ChIP-Seq signals measured in WT or GPS2-KO MCF-7 cells.

The analysis of HDAC3 ChIP-Seq data in WT cells identified 3,569 significant chromatin associations (Fig. 2*A*). Among them, 2,039 (57.13%) overlapped with a GPS2 peak, indicating regulatory regions co-occupied by the two factors, which is in accord with these two factors being integral components of the same complex (Fig. 2*A* and Dataset S1*B*). As previously reported for other components of the NCoR/SMRT complex, the genomic distribution of HDAC3 peaks showed a prevalence of intronic and intergenic binding, with no significant differences if dividing the regions based on the co-occupancy with GPS2 (Dataset S1*C*). Based on integration with the MCF-7 chromatin states predicted for WT cells (46), we identified 787 HDAC3 peaks that overlapped with regions classified as enhancers, again with no significant differences in the respective representation of GPS2 overlapping and nonoverlapping peaks (Dataset S1*C*).

In accord with previous studies showing both GPS2 alone and the NCoR/SMRT corepressor complex as a whole being recruited by a number of different transcription factors (TFs), motif enrichment analysis showed an enrichment for several classes of TFs. De novo analysis revealed an enrichment for ZNF384, TFAP2C, and PAX9 motifs among the HDAC3-GPS2 overlapped peaks, and binding motifs for bHLH, homeobox, zing fingers factors, and nuclear receptors were identified by HOMER across both common and separate peaks (Dataset S1 *E* and *F*). Interestingly, dividing the HDAC3 peaks into four quartiles (Q1-Q4) (Fig. 2*B*) based on ChIP-Seq enrichment significance revealed a specific enrichment for p53 and PAX5 (Q1) and nuclear receptor binding sites (Q2) among peaks with higher HDAC3 binding, whereas the peaks with lower binding intensity are enriched for Zf (Q3) and bHLH (Q4) binding sites (Dataset S1*F*). In accord with these differences in TFs binding sites, metabolism-related terms [e.g., *acyl-CoA metabolic process (GO:0006637)*, *fructose 6-phosphate metabolic process (GO:0006002)*, *cellular glucose homeostasis (GO:0001678),* and *glycerophospholipid biosynthetic process (GO:0046474)*] were enriched among high-intensity HDAC3 peaks (Q1 or Q2) when considering the genes annotated within 10 Kbp from the HDAC3 peak (Fig. 2*C* and Dataset S1*D*). Conversely, Q3 and Q4 HDAC3 peaks were enriched in terms related to cell cycle control [e.g., *positive regulation of cell cycle G1/S phase transition (GO:1902808)* and *regulation of metaphase plate congression (GO:0090235)*] and developmental processes [e.g., *regulation of transforming growth factor beta2 production (GO:0032909)* and *positive regulation of cell differentiation (GO:0045597)*] (Fig. 2*C*). Unexpectedly, GPS2 appears to bind most strongly to the regions with weak HDAC3 signal as a higher proportion of GPS2 peaks was observed in low-intensity HDAC3 peaks (Q4) (*P* < 0.0001) in comparison to higher-intensity HDAC3 peaks (Q1, Q2, and Q3), whereas HDAC3 binding is stronger in regions with weak GPS2 signal (Fig. 2*D*). These results together suggest that different TFs may be recruiting the two factors through a different stoichiometry. Nonetheless, the loss of GPS2 appears to affect weak and strong HDAC3 in a similar manner as we observed a striking decrease in HDAC3 recruitment to chromatin in GPS2-KO cells across all the quartiles, with more than 90% of the HDAC3 peaks (3,304 over 3,569) found decreased in GPS2-KO cells, 2,364 of which were significantly (*P* < 0.05) decreased (Fig. 2 *D* and *E* and Dataset S1*G*).

### Transcriptome Analysis of MCF-7 Cells in the Absence of GPS2.

**Fig. 3.**
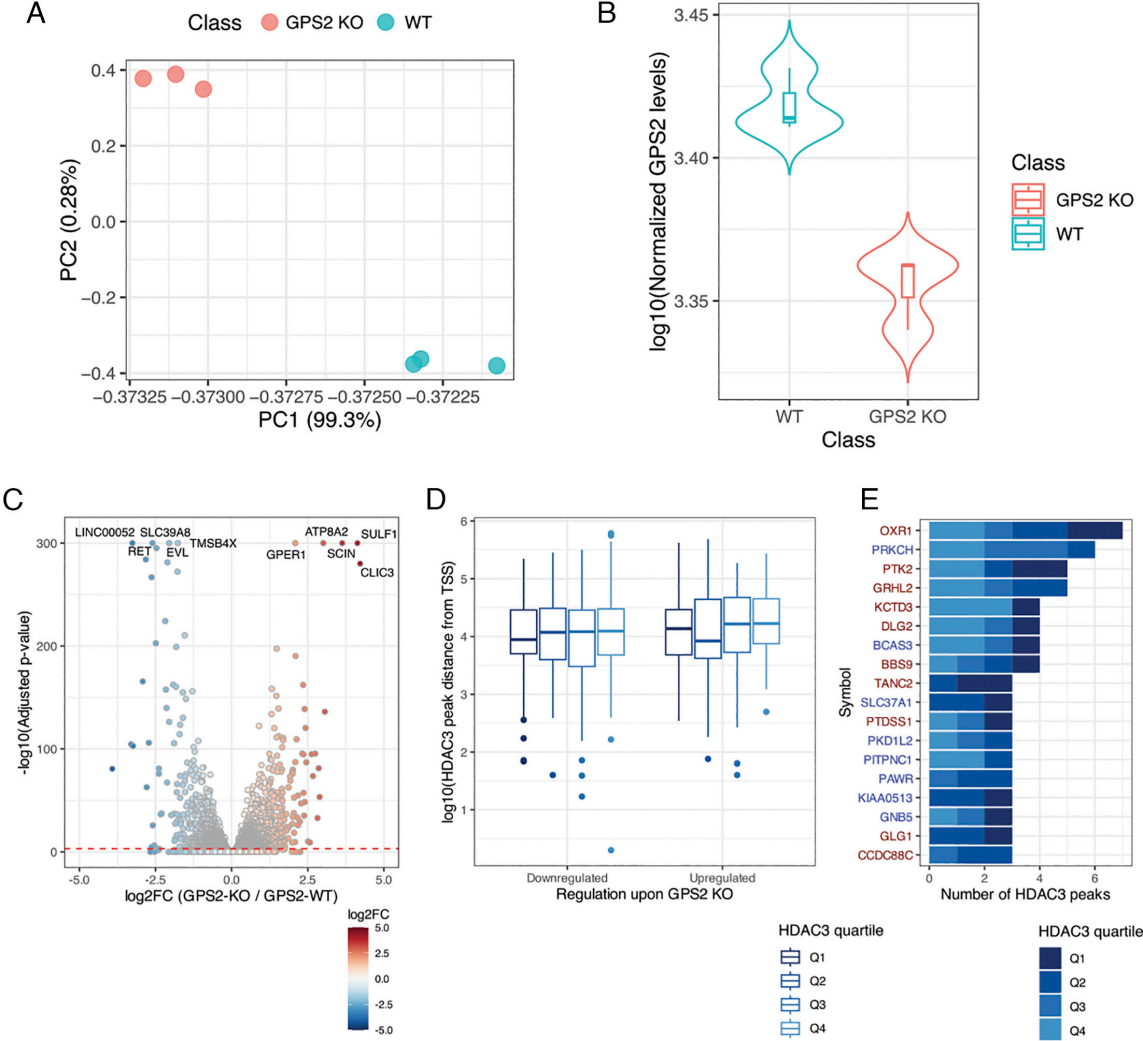
(*A*) Representation of the PCA results of the gene expression levels in WT and GPS2-KO MCF-7 cells. (*B*) Violin plot of the normalized GPS2 levels in WT and GPS2-KO cells. (*C*) Volcano plot representing the results of the differential expression analysis between WT and GPS2-KO cells. The identifiers of the five upregulated and downregulated genes associated with the highest statistical significance are reported. (*D*) Boxplot of the distance between HDAC3 peak centers and DEG TSSs. The HDAC3 peaks were stratified based on the quartile of HDAC3 ChIP-Seq signals in WT cells. (*E*) Bar plot of the number of HDAC3 peaks in each peak quartile localization within 10 kp from a DEG TSS. The figure reports the DEGs associated with the highest number of HDAC3 peaks. The gene symbol is color-coded based on the gene differential expression in GPS2-KO cells (red, upregulation; blue, downregulation).

Previous studies revealed that HDAC3 contributes to both transcriptional repression and activation via deacetylation of distinct protein targets (6, 11, 47). Based on the striking decrease in HDAC3 binding to most genomic locations recorded by ChIP-Seq, we predicted that loss of GPS2 would equally impair the expression of HDAC3-repressed and -activated genes. To investigate the transcriptional effect of GPS2 deletion, total RNA sequencing (RNA-Seq) of GSP2-KO and WT cells was performed on GPS2-WT and GPS2-KO MCF-7 cells in triplicate (48). PCA analysis showed that the expression profiles of the three replicates of the GPS2-KO experiments differed from those of WT cells (Fig. 3*A*). As expected, GPS2 levels significantly decreased in the GPS2-KO cells (adj. *P* < 0.05) (Fig. 3*B*). By differential expression analysis, 3,744 genes were observed as significantly differentially expressed (DEG, adj. *P* < 0.001), of which 1,837 were down- and 1,907 were up-regulated (Fig. 3*C* and Dataset S2*A*). Most significantly regulated genes include targets of ER regulation and genes previously associated with breast cancer progression and poor outcomes, including upregulation of *SULF1*, *SCIN*, *ATP8A2*, *GPER1,* and *CLIC3* (49–53) (Dataset S2*A*). Functional enrichment analysis of all DEGs showed an enrichment of terms related to transcriptional regulations, cell cycle, and DNA damage response for the up-regulated genes. Conversely, down-regulated genes were enriched for ribosome biogenesis and inflammatory response-related processes (Dataset S2*B*). To focus on the transcriptional outcome of HDAC3 dismissal in the absence of GPS2, the analysis was focused on 455 DEGs (219 up-regulated and 236 down-regulated) associated with at least one HDAC3 peak (Dataset S2C). In total, 681 HDAC3 peaks were associated with these genes, and among them, a comparable distance from gene TSS was observed between the up- and down-regulated genes (*P* = 0.352, Fig. 3*D*). Similarly, no significant differences were observed in the proportion of HDAC3 peaks from different quartiles mapped in the proximity of up- and down-regulated genes (chi-square *P* = 0.83). Among the 681 HDAC3 peaks, 639 (93.8%) were associated with a ChIP-Seq signal decreasing in GPS2 KO cells with a comparable proportion between up- and down-regulated genes (324 vs. 315 peaks, respectively, chi-square *P* = 1). Considering the peaks associated with the highest number of HDAC3 peaks within a distance of 20 Kbp from their TSS, 18 DEGs were associated with more than two peaks and included *OXR1* (seven peaks), *PRKCH* (six peaks), and *PTK2* and *GRHL2* (five peaks each) (Fig. 3*E*). Functional analysis of the HDAC3 peak-associated genes showed that those up-regulated in GPS2 KO cells were enriched in terms related to protein localization within the cells, Fc-gamma receptor signaling, regulation of hormone, and sphingolipid metabolism (Dataset S2*B*). Conversely, GPS2-KO-down-regulated genes were enriched in protein transport, response to viral infection, and cell differentiation. Similar enrichments were observed by focusing the analysis on DEGs characterized by a promotorial HDAC3 peak within 2 kbp (n = 109; 49 up- and 60 down-regulated) (Dataset S2*B*). Motif enrichment analysis showed a difference in motifs predicted in these peaks with NR6A1 and SCRT2 motifs enriched in upregulated genes and ONECUT1 and ZNF460 binding sites enriched for the downregulated genes (Dataset S1*C*).

To further evaluate the dual regulatory activity of HDAC3 in gene repression and activation, the set of identified DEGs was overlapped with the subset of *Drosophila Melanogaster* genes dysregulated upon HDAC3 KO (GSE206247) (54). This analysis highlighted 50 and 26 DEGs that were respectively down-regulated and up-regulated in both studies (Dataset S2*D*). Upregulation of Myc targets *ZFP36L1* and *ALDH9A1*, together with the significant upregulation of MYC expression observed by RTqPCR (Fig. 1*D*) and the below-threshold increase in gene expression observed by RNA-Seq with respect to the control condition (log2FC = 0.11, *P* = 0.02 and adj. *P* = 0.06), suggest a derepression of MYC signaling in the absence of GPS2. On the contrary, downregulation of mitochondrial genes *OAT, MTHFS,* and *ALDH7A1* is in accord with previous studies reporting critical activator roles for both GPS2 and HDAC3 in regulating mitochondrial gene expression despite their well-characterized function as corepressors of nuclear-receptor-mediated transcription (11, 14, 29). To further explore the impact of GPS2 deletion on mitochondrial gene expression in MCF-7 cells, we overlapped the DEGs identified by RNA-Seq in MCF-7 cells with the genes listed by MitoCarta 3.0 (55). Out of 1,146 neMITO included in this dataset, we found 168 significantly downregulated genes in GPS2-KO cells (Dataset S2*D*). Three were characterized by HDAC3 and GPS2 cobinding to the promoter (*PTCD1, MCRIP2,* and *THEM4*), and nine had a proximal HDAC3 peak. HDAC3 recruitment to most of them was impaired in the absence of GPS2. At the same time, we observed that 124 neMITO genes were up-regulated in GPS2 KO cells. Among them, three *UQCRC1, DECR1,* and *ACP6* were marked by an HDAC3 peak at the promoter and four by a proximal HDAC3 peak. HDAC3 recruitment to all was impaired in the absence of GPS2. Pathway analysis shows a prevalence of general mitochondria-related terms enriched among the down-regulated genes (adj. *P* < 0.001), like *Mitochondrion Organization (GO:0007005)*, *Mitochondrial Transport (GO:0006839)*, and *Mitochondrial Gene Expression (GO:0140053)* (Dataset S2*E*). In contrast, up-regulated genes were enriched in more specific processes like *Quinone Biosynthetic Process (GO:1901663)*, *Branched-Chain Amino Acid Metabolic Process (GO:0009081)*, and *Heme Biosynthetic Process (GO:0006783).* Together, these results indicate that GPS2 is critical to prevent HDAC3 ubiquitination and license its recruitment to numerous chromatin locations across the genome, with GPS2 deletion equally impacting genes repressed and activated by HDAC3.

### GPS2-Mediated Regulation of TAB2 Ubiquitination.

**Fig. 4.**
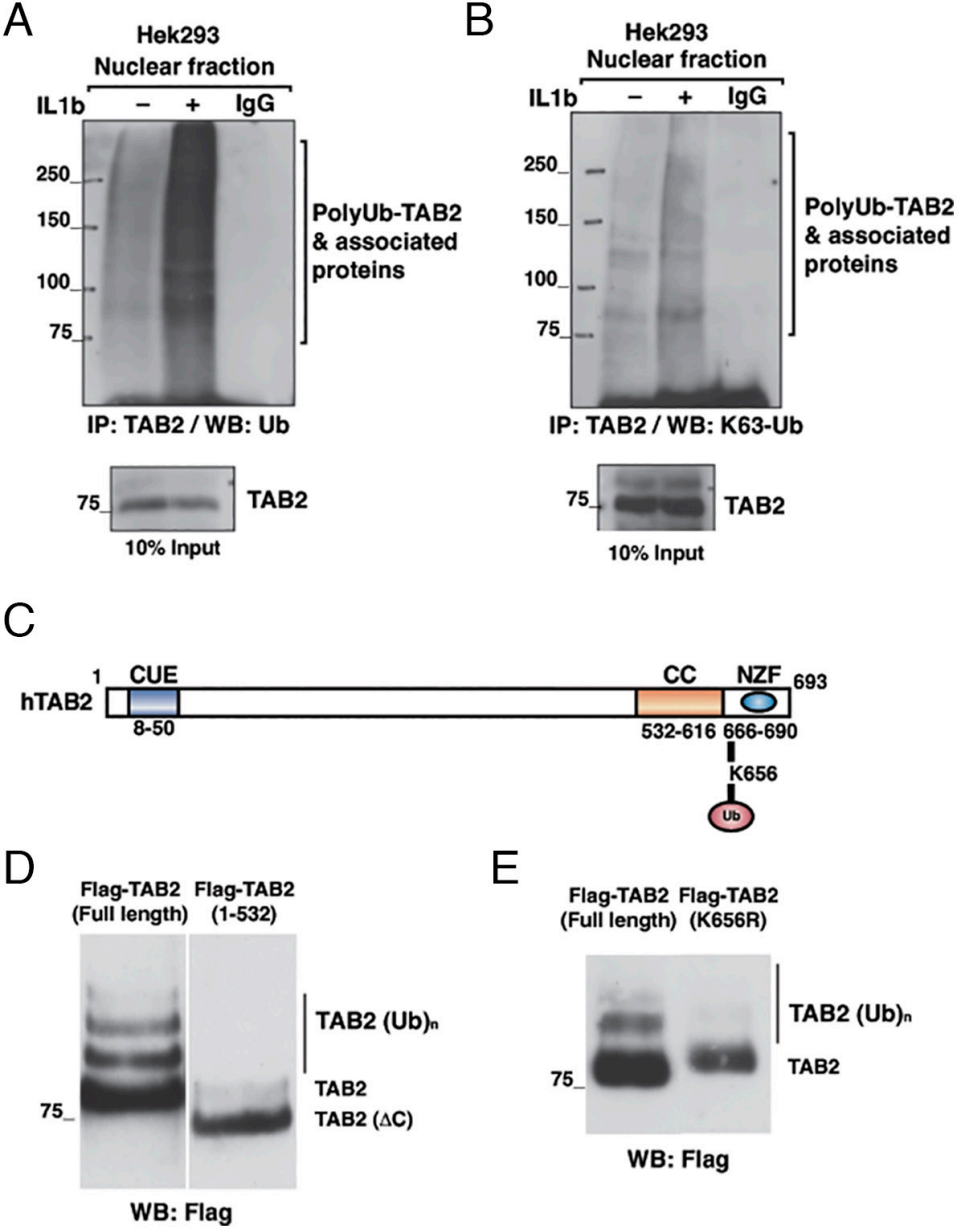
(*A*) IL-1β-dependent ubiquitination of TAB2. HEK293T cells were treated with IL-1β for 5′ and fractionated into cytoplasmic and nuclear extracts. The nuclear fraction was immunoprecipitated with anti-TAB2 antibody and subjected to western blot with anti-ubiquitin antibody. (*B*) Same as (*A*). Western blot with an antibody-specific K63 polyubiquitin chains. (*C*) Schematic representation of human TAB2 (modified from ref. 60) with identified ubiquitination site. (*D*) HEK293T cells were transfected with FLAG-TAB2-wt and FLAG-TAB2-DeltaC plasmid, treated with IL-1β for 5 min, and fractionated into cytoplasmic and nuclear extracts; nuclear fraction was immunoprecipitated with anti-flag antibody and subjected to western blot with anti-flag antibody. (*E*) Same as (*D*). Here, HEK293T cells were transfected with FLAG-TAB2-wt and FLAG-TAB2-K656R plasmid.

The results shown above indicate that GPS2 regulates both chromatin occupancy and K63 ubiquitination status of at least one component of the NCoR/SMRT complex. To explore whether the ubiquitination of other factors within the NCoR/SMRT complex may be affected by the lack of GPS2, possibly contributing to aberrant gene regulation in GPS2-KO cells, we explored the GPS2-regulated K63 ubiquitome that we recently characterized in mouse embryonic fibroblasts and breast cancer MDA-MB231 cells (35). This analysis revealed that TAB2, a facultative component of the NCoR/SMRT complex, was among the top 25 candidates isolated by binding to the K63Ub-binding domain V3K0 and found upregulated in GPS2-KO cells as compared to the WT parental line (35). Because increased immunoprecipitation of TAB2 upon enrichment for K63 ubiquitinated proteins could be indicative of either more ubiquitination of TAB2 itself and/or increased association to other ubiquitinated targets, we turned to HEK293T cells to confirm that TAB2 is indeed ubiquitinated and identify the ubiquitination site/s by liquid chromatography-tandem mass spectrometry (LC–MS/MS). Because TAB2 was shown to play a key role in IL-1β-dependent derepression of ERα-target genes (56–58), and TRAF6, the ubiquitin ligase responsible for HDAC3 modification, is also known to mediate TAB2 ubiquitination upon IL-1β stimulation (59), we decided to focus on IL-1β-mediated ubiquitination events and ask whether localized ubiquitination of TAB2 by TRAF6 played a role in the dismissal of the NCoR/SMRT complex from derepressed genes. To begin, we confirmed that the amount of K63 ubiquitin chains associated with endogenous TAB2 in the nuclear compartment of HEK293T cells rapidly increased upon IL-1β stimulation (Fig. 4 *A* and *B*). Next, we immunoprecipitated FLAG-TAB2 from nuclear extracts of IL-1β-treated HEK293T cells and by LC–MS/MS identified Lysine 656 as the only ubiquitinated residue following IL-1β treatment (Fig. 4*C*). To further confirm this result, we used a dual approach. First, we compared the migration pattern of Flag-TAB2 full length with that of Flag-TAB2 with a C-terminal deletion encompassing K656 (Fig. 4*D*). Second, we removed the putative ubiquitination site by substituting the Lys in position 656 with an Arg via site-directed mutagenesis (Flag-TAB2_K656R_) (Fig. 4*E*). As expected, in both instances, removal of K656 alone or within the C-terminal domain was sufficient to eliminate the presence of the HMW bands that were observed upon IL-1β treatment, thus confirming that nuclear TAB2 is ubiquitinated in response to IL-1β treatment on K656.

### Regulation of the NCoR/SMRT Complex by TRAF6.

**Fig. 5.**
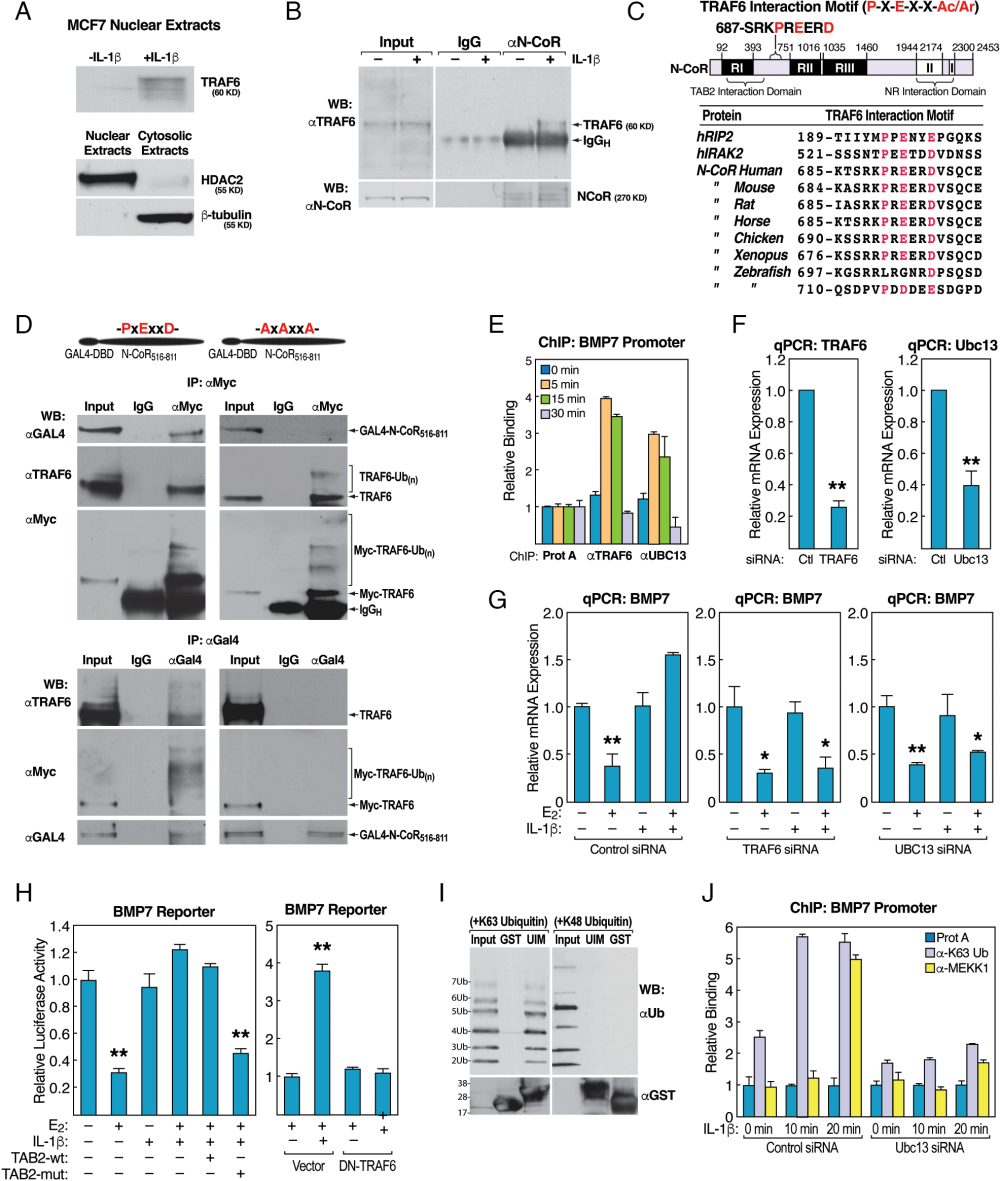
(*A*) Western blotting with anti-TRAF6 Ab to visualize the nuclear translocation of TRAF6 in MCF-7 cells treated with IL-1β for 5 min. WBs for HDAC2 and β-tubulin were run on the cytoplasmic and nuclear extracts as a control. (*B*) TRAF6 interaction with NCoR in IL-1β-treated MCF7 cells by immunoprecipitation with anti-NCoR Ab. (*C*) Graphic representation of the NCoR protein showing the putative TRAF6 interaction motif, and sequence alignment of similar motifs found in human RIP2, IRAK2, and NCoR across species. (*D*) Interaction between TRAF6 and the identified interaction motif in HEK293T cells transfected with Myc-TRAF6 and wild type (*Left*) or PxExxDAxAxxA mutant (*Right*) GAL4-NCoR_516-811_ plasmids. Immunoprecipitation was performed either with anti-Myc, anti-GAL4, or control IgG, and blotted with anti-GAL4, anti-TRAF6, or anti-Myc Ab. (*E*) ChIP experiment showing rapid and transient recruitment of TRAF6 and Ubc13 to the *BMP7* promoter in response to IL-1β in MCF7 pretreated with E_2_ for 1 h. (*F*) Validation of siRNA efficiency in MCF7 cells. Cells are transfected with siRNA (siCtl, siTRAF6, or siUbc13) for 48 h and relative expression measured by RTqPCR, with normalization to *b-actin.* (*G*) BMP7 derepression by IL-1β, as measured by RT-PCR, requires both TRAF6 and Ubc13. MCF7 cells transfected with either control, TRAF6, or Ubc13 siRNA, were treated with E_2_ and/or IL-1β for 6 h. (*H*) Overexpression of dominant-negative TRAF6 or mutated TAB2 abrogates derepression by IL-1β in U2OS-ERα cells transiently transfected with BMP7_promoter_ reporter and treated with E_2_ and vehicle (PBS) or IL-1β for 36 h. Luciferase activity was assayed and normalized to b-gal activity. (*I*) MEKK1’s ubiquitin-interacting motif (UIM) interacts with K63-linked, but not K48-linked, polyubiquitin chains. GST and GST-MEKK1-UIM proteins were expressed in bacteria, purified, and used for GST pulldown with recombinant K63- (*Left*) or K48-linked (*Right*) polyubiquitin chains, followed by western blotting with anti-ubiquitin or anti-GST Ab. (*J*) Ubc13 is required for accumulation of K63-linked polyubiquitin chains and for the recruitment of MEKK1 to the *BMP7* promoter in MCF7 cells transfected with control or Ubc13 siRNA and pretreated with E_2_ for 1 h prior to treatment with IL-1β. ChIP was performed with anti-K63-ubiquitin or anti-MEKK1 Ab or protein A beads alone as control. Data are represented as mean ± SEM. *P < 0.05, **P < 0.01 vs. vehicle control, Student’s *t* test.

Together, these results suggest that GPS2-mediated inhibition of corepressors ubiquitination may be used for modulating gene expression at the level of specific target genes. As both TAB2 and HDAC3 are targets of the E3 ubiquitin ligase TRAF6, we investigated whether TRAF6 contributed to this regulatory strategy, which would require TRAF6 translocation to the nucleus and a mechanism for regulating its recruitment to regulatory units bound by the NCoR/SMRT complex. First, we addressed TRAF6 subcellular localization in IL-1β treated MCF-7 cells. In accord with our hypothesis, TRAF6 was detected in the nuclear compartment within 5 min of treatment with IL-1β by immunoblotting of fractionated cytosolic and nuclear extracts (Fig. 5*A*). Moreover, by coimmunoprecipitation, we detected an IL-1β-dependent interaction between endogenous TRAF6 and NCoR in nuclear extracts from MCF-7 cells treated with IL-1β (Fig. 5*B*). To identify the possible molecular surface mediating this recruitment, the various components of the corepressor complex were explored in search of putative TRAF6 interaction domains. A TRAF6 consensus interaction sequence was previously reported in cytokine receptors and signal transduction proteins, consisting of a proline residue followed in order by any amino acid, a glutamic acid residue, any two amino acids, and an acidic or aromatic residue (PxExxAc/Ar) (61). Sequence analysis of NCoR revealed a perfect consensus motif (PREERD) at aa 690 to 695, adjacent to the TAB2-interacting region and the HDAC3-binding domain. The motif is evolutionarily conserved in species tracing back to *Xenopus*, and a similar motif is present in zebrafish NCoR (Fig. 5*C*). To demonstrate the functional relevance of this conserved motif in mediating TRAF6 interaction with the complex, we mutated the PREERD motif in an NCoR fragment spanning aa 516 to 811 by site-specific mutagenesis (PxExxD was converted to AxAxxA). As predicted, the interaction between TRAF6 and NCoR by immuno-precipitation was observed only when using wild type (PxExxD) NCoR_516-811_, but not the mutated fragment (Fig. 5*D*).

To address the functional role of TRAF6-mediated events on localized gene targets, we then focused on *BMP7*, an estrogen receptor target gene previously shown to be derepressed upon IL-1β treatment due to TAB2-dependent dismissal of the NCoR/SMRT complex (57) and asked to which extent TAB2 ubiquitination contributes to IL-1β-mediated dismissal of the NCoR/SMRT complex. First, by ChIP analysis, it was confirmed that both TRAF6 and its partner E2 enzyme, Ubc13, were detected bound to the *BMP7* promoter at 5 and 15 min after IL-1β stimulation of E_2_-treated MCF-7 cells (Fig. 5*E*). Then, we asked to which extent K63 ubiquitination by Ubc13/TRAF6 contributed to IL-1β-induced dismissal of the NCoR/SMRT complex. Our results indicate that preventing K63 ubiquitination by transient downregulation of either TRAF6 or Ubc13 was sufficient to impair *BMP7* derepression by IL-1β as measured by RTqPCR (Fig. 5*F*). Similarly, overexpression of a dominant-negative TRAF6 construct or the Flag-TAB2_K656R_ mutant inhibited the IL-1β-dependent derepression of a luciferase reporter construct driven by the *BMP7* promoter (Fig. 5 *G* and *H*). Together, these data confirm that K63 ubiquitination of TAB2 by TRAF6/Ubc13 plays a critical role in promoting the derepression of *BMP7* upon IL-1β stimulation.

Finally, we investigated the mechanism by which TRAF6/Ubc13-mediated K63 ubiquitination regulates *BMP7* derepression. Previous studies indicated that dismissal of the NCoR/SMRT complex from the *BMP7* promoter depends on the recruitment and the action of the Mitogen-activated protein kinase kinase kinase 1 (MAP3K1/MEKK1) (57, 58). As MEKK1 contains two overlapping ubiquitin-interacting motifs (UIM), we hypothesized that TRAF6/Ubc13-mediated ubiquitination of one or more targets may be important for MEKK1 recruitment. In accordance with this hypothesis, in vitro Glutathione S-transferase (GST) pull-down assays showed that the UIM region of MEKK1 (aa 1,137 to 1,194) specifically interacts with recombinant K63-linked polyubiquitin chains, and not with K48-linked chains (Fig. 5*I*). Most importantly, ChIP analysis confirmed that the accumulation of K63 ubiquitin chains on the *BMP7* promoter upon IL-1β treatment precedes the recruitment of MEKK1, and that blocking ubiquitin accumulation by Ubc13 knockdown, prevented recruitment of MEKK1 (Fig. 5*J*), thus confirming that localized ubiquitination events on target promoters can have a profound effect on transcriptional regulation through direct and indirect effects on the recruitment/dismissal of regulatory factors and chromatin remodeling enzymes.

## Discussion

**Fig. 6.**
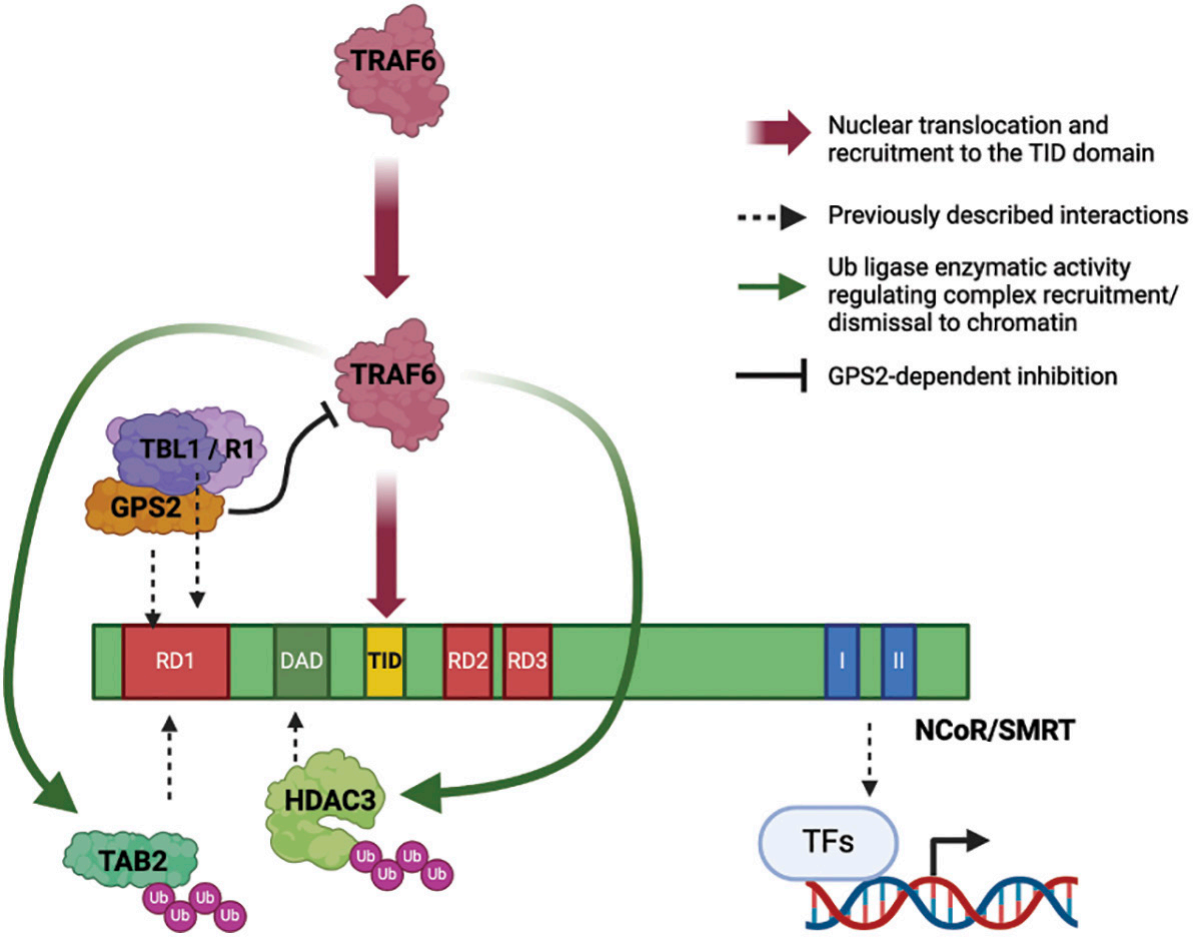
Graphical abstract representation of TRAF6 nuclear translocation and recruitment to the TRAF-interaction domain (TID) on NCoR/SMRT in response to IL-1β (red arrows). Within the NCoR/SMRT complex, GPS2 maintains TRAF6 activity under negative regulation (black solid line), while HDAC3 and TAB2 were identified as targets of TRAF6-mediated ubiquitination (green arrows). Dotted black arrows represent previously reported interactions between components of the NCoR/SMRT corepressor complex and associated TFs. RD1, RD2, and RD3 represent repressor domains; DAD is the deacetylase activation domain, while interaction with recruiting TFs occurs through the C’-terminal nuclear receptor–interacting domains I and II.

G-Protein Suppressor 2 (GPS2) has emerged in recent years as a critical regulator of inflammation and metabolism (19–21, 23, 25–29, 31–35). A growing body of literature indicates that this is achieved through complementary genomic and nongenomic functions that are dependent, at least in part, on GPS2 ability to inhibit nonproteolytic ubiquitination by the E2 ubiquitin-conjugating enzyme Ubc13 (25–29). In the nucleus, it is well appreciated that GPS2 regulates gene expression by acting both as a corepressor and a coactivator for numerous TFs (16, 19, 23, 25, 29, 31, 33, 34, 62). However, the underlying molecular mechanisms are not fully understood, and whether ubiquitin regulation constitutes a common thread across the different transcriptional roles played by GPS2 remained unclear. Here, we focused on GPS2 role within the NCOR/HDAC3 corepressor complex and found its presence to be crucial for counteracting the destabilizing effect triggered by TRAF6/Ubc13-mediated ubiquitination of other components of the complex, including the histone deacetylase HDAC3 and the facultative adaptor TAB2 (Fig. 6).

HDAC3 is a class I histone deacetylase and the major chromatin remodeling enzyme associated with the NCoR/SMRT corepressor complex (10). Its activity is responsible for histone deacetylation over repressed target genes but has also been associated with the deacetylation of nonhistone targets in the context of activated genes. Our results indicate that GPS2 deletion in MCF-7 breast cancer cells leads to increased HDAC3 ubiquitination and dismissal from most of its genomic locations, including both repressed and activated gene targets. This suggests that restriction of ubiquitination activity may be critical to prevent breast cancer cell growth and metabolic reprogramming, as GPS2 deletion is associated with increased expression of the *MYC* gene and altered expression of genes involved in cancer progression, metastatic potential, mitochondrial functions, and lipid metabolism. This is in accord with previous studies showing upregulation of *MYC* in hepatocellular carcinoma through dissociating K63-ubiquitinated HDAC3 from the *MYC* promoter (41). Altered MYC is implicated in various cancer types, including breast cancer, where it is associated with poorer outcomes (63–65). As our previous studies in triple-negative breast cancer cells indicated that loss of GPS2 was associated with a more aggressive phenotype due to hyperactivation of PI3K/AKT signaling (43), these results suggest that the concurrent upregulation of PI3K and MYC signaling, due to unrestricted ubiquitination of cytosolic and nuclear targets, may be contributing to the observed phenotype of GPS2-null cells.

Within the NCoR/SMRT complex, GPS2-mediated regulation is not restricted to HDAC3 but is also important for preventing the ubiquitination of TAB2, a facultative adaptor previously shown to play a key role in promoting the derepression of target genes upon proinflammatory stimulation (57, 58). Our study reveals that TAB2 ubiquitination upon IL-1β stimulation is mediated by the E3 ubiquitin ligase TRAF6, which rapidly translocates to the nucleus upon IL-1β stimulation and is recruited to regulated genes via direct interaction with NCoR. TRAF6 serves as a key hub for several breast cancer-driver signal transduction pathways, including PI3K/AKT/mTOR, Toll-like receptor (TLR), mitogen-activated protein kinase (MAPK), and Ras/Src Family Kinases, with downstream effects on NFκB and AP-1 signaling (66–69). This study adds to the literature concerning TRAF6 role in signal transduction by showing that TRAF6-mediated regulation of gene expression in response to pro-oncogenic and inflammatory signals is not only achieved via modulation of cytosolic signaling (61, 70–72) but also through direct regulation of transcriptional events in the nucleus, with GPS2 establishing a critical checkpoint on both processes. Intriguingly, TRAF6 translocation to the nucleus has also been observed upon insulin stimulation, with TRAF6 nuclear activity being required for K63 ubiquitination of AKT1, which is also negatively regulated by GPS2 (28, 73). As insulin-induced phosphorylation of NCoR by AKT in the liver provides a switch for metabolic genes regulated by different nuclear receptors, the complex interplay between the cytosolic and nuclear signaling events regulated by TRAF6 appears central to responding to proinflammatory and metabolic signals through modulation of corepressor presence and/or transcriptional activity. Indeed, our results indicate that TRAF6-mediated ubiquitination of TAB2 contributes to MEKK1 recruitment, thereby licensing the promoter for corepressor clearance upon IL-1β stimulation. At the same time, previous studies show that TRAF6 activation by RANK/TLR4 plays a key role in facilitating HDAC3 conversion from corepressor to coactivator by initiating a bifurcated kinase cascade that includes TRAF6-ERK1 signaling promoting the formation of an NCoR/HDAC3/PGC1β complex and TRAF6-TBK1 signal enhancing HDAC3 activity necessary for PGC1β deacetylation (6). By elucidating the mechanism of TRAF6 recruitment to chromatin through direct interaction with NCoR/SMRT and identifying GPS2 as a key regulator of TRAF6 activity toward the NCoR complex, our findings expand our understanding of the importance of proinflammatory signaling and K63 ubiquitination in modulating both the recruitment to chromatin and the functional outcome of HDAC3 activity.

Crosstalk between transcriptional and posttranslational regulatory events is also observed in the case of *MYC*, as TRAF6 regulates *MYC* expression via HDAC3 ubiquitination while also inhibiting c-Myc acetylation on Lys148 via ubiquitination of the same residue (74). As Lys148 acetylation positively regulates genes involved in the mitotic apparatus while inhibiting the expression of p53 and cytokine pathways (75), combined effects of TRAF6 activation on *MYC* upregulation and transcriptional activity may contribute to altered transcriptomics of GPS2-KO cells, with these events adding to the transcriptional changes driven by reduced HDAC3 occupancy.

In conclusion, dissecting the impact of GPS2 deletion in breast cancer cells has highlighted the central role of the TRAF6/Ubc13 ubiquitin machinery in coordinating signaling pathways across subcellular compartments and revealed that the dysregulation of ubiquitination could represent a critical factor in cancer growth and metabolic adaptation. In this light, modulation of ubiquitination activity by GPS2 can function as a key determinant in controlling cancer progression, thus underscoring its potential as a targeted therapeutic avenue for intervention.

## Materials and Methods

### Cell Lines.

MCF-7 cells purchased from American Type Culture Collection were cultured in high glucose Dulbecco’s modified Eagle’s medium (DMEM) with 10% fetal bovine serum. GPS2-KO MCF-7 cells were generated in-house by CRISPR/Cas9-mediated genomic recombination using the same design developed for MDA-MB231 cells (43). Specifically, CRISPR-Cas9 genome editing was performed using two sets of single guide RNA (sgRNAs) targeting exons 2 and 6 of the human *GPS2* sequence. The two sets of sgRNAs were cloned separately into the LentiCRISPRv2 lentiviral vector (Kindly shared by Feng Zhang, Addgene plasmid #52961). 3.5 mg of each viral plasmid was cotransfected with packaging plasmids pCMV-VSV-G and psPAX2 (Addgene plasmids #8454 and #12260) into HEK293T cells using Lipofectamine 3000. Media were changed after 24 h, and viral supernatants collected at 60 h. The virus was filtered through a 0.46 μm low protein binding filter membrane and used immediately or stored at −80 °C for later use. Transduction of target cell lines was performed in a six-well plate with 1 to 1.5 mL of viral supernatant and 8 μg/mL polybrene in DMEM with 10% FBS. Media were changed after 24 h, and cells split into selection medium (puromycin 2.5 μg/mL) at 48 h. Surviving heterogeneous pools were controlled for GPS2 deletion by targeted PCR and western blotting.

### Chemicals and Antibodies.

Anti-GPS2 C-terminal (aa 307 to 327) antibody was previously generated in rabbit (29). Anti-NCoR antibody was previously generated in rabbit (76). Commercial antibodies used for western blotting, immunoprecipitation, and ChIP experiments include HDAC3 rabbit polyclonal antibody #ab7030 (Abcam); anti-HDAC3(H-99) rabbit polyclonal antibody #sc-11417 (Santa Cruz Biotechnology); anti-ubiquitin mouse monoclonal antibody (P4D1 clone, Cell Signaling Technology); anti-β-tubulin mouse monoclonal antibody #T0198 (Sigma-Aldrich); anti-HDAC2 rabbit polyclonal antibody #ab16032 (Abcam); anti-TAB2 mouse monoclonal antibody sc-398188, goat polyclonal antibody sc-11850, and rabbit polyclonal sc-20756 (Santa Cruz Biotechnology); anti-K63 ubiquitin rabbit monoclonal antibody #05-1308 (Millipore); anti-TRAF6 rabbit polyclonal antibody sc-7221 (Santa Cruz Biotechnology); anti-Ubc13 rabbit polyclonal antibody #10243-1-AP (ProteinTech); anti-Ubc13 (131-148) rabbit polyclonal antibody #PA1-41188 (Thermo Scientific); anti-FLAG mouse monoclonal antibody, clone M2, #F3165, and anti-Flag-HRP #A8592 (Sigma-Aldrich); anti-MYC(9E10) mouse monoclonal antibody #sc-40 (Santa Cruz Biotechnology); and anti-GST(56C1) mouse monoclonal antibody #sc-80998 (Santa Cruz Biotechnology).

The following small interfering RNA (siRNA) were obtained from Santa Cruz Biotechnology and used for silencing via transient transfection with Lipofectamine 2000 (10 nM final concentration): TRAF6 (sc-36717), Ubc13 (sc-43551), and control (sc-37007). Treatments with human IL-1β, purchased from Calbiochem, were performed at a final concentration of 10 ng/mL for the time indicated in each experiment.

### Protein Extracts and Western Blotting.

Cells were lysed with 10 mM HEPES pH 7.9, 1 mM Ethylenediaminetetraacetic Acid (EDTA), 210 mM Mannitol, 70 mM Saccharose, 1× protease inhibitor cocktail (PIC) (Roche), 10 mM N-Ethylmaleimide (NEM), passed through a 25G syringe and centrifuged for 10 min at 4 °C (2,000× g). The cytoplasmic fraction was further cleared for 10 min at 4 °C (16,500× g). The nuclear pellet was resuspended in 20 mM Tris-HCl pH 8.0, 25% glycerol, 420 mM NaCl, 1.5 mM MgCl_2_, 0.2 mM EDTA, 0.1 mM phenylmethylsulfonyl fluoride, 0.5 dithiothreitol (DTT), 1× PIC, and 10 mM NEM and centrifuged for 10 min at 4 °C (max speed) to clear the lysate. Proteins were quantified by Bradford, resolved by Sodium Dodecyl Sulfate Polyacrylamide Gel Electrophoresis, and analyzed by immunoblotting using Imagine Lab Software 6.0.1 (Bio-Rad Laboratories, Inc.).

### Immunoprecipitation of Ubiquitinated Proteins.

K63 ubiquitinated proteins were isolated using the Vx3K0 ubiquitin-binding domain as described in ref. 35. The His-tagged K63-Super-UIM (Vx3K0) was amplified from plasmid pQCXIP-Vx3K0-mEGFP [(77); Addgene #35527] and inserted into pET28a. His-tagged Vx3K0 protein was overexpressed in *Escherichia coli* BL21(DE3) cells, upon induction with 0.5 mM isopropyl-β-D-thiogalactoside for 4 h at 30 °C. Harvested cells were lysed by sonication in lysis buffer (50 mM NaH_2_PO_4_, 500 mM NaCl, 0.1% SDS, 1% Triton X-100, and 10% glycerol). According to the manufacturer’s instructions, his-tagged Vx3K0 protein was purified from cell lysates using Ni-NTA agarose (Invitrogen, USA). After washing in 50 mM NaH_2_PO_4_, 500 mM NaCl, 0.5% Triton X-100, 10% glycerol, and 20 mM imidazole, the His-tagged Vx3K0-conjugated agarose was stored at 4 °C in phosphate-buffered saline (PBS) supplemented with 30% glycine.

### RT-PCR Analysis.

RNA was isolated from cells following the manufacturer protocol for the RNeasy Kit (QIAGEN). First-strand complementary DNA (cDNA) synthesis from the total RNA template was performed with an Iscript cDNA Synthesis System (Bio-Rad). PCRs were conducted on a ViiA7 Real-Time PCR System, using the SYBR Green method and Human Cyclophilin A RNA as a normalizing target.

### ChIP Assay.

ChIP assay was performed following established protocols (9, 25, 78). In brief, MCF-7 cells were cross-linked with formaldehyde 1% for 10 min at room temperature, lysed, and sonicated for two cycles of 10 min (max power 30 s on, 30 s off) to reduce DNA length to between 200 and 500 bp. Cross-linked material was immunoprecipitated with 1 to 5 μg of the specific antibody overnight at 4 °C and followed by incubation with protein A-sepharose beads for 1 h to collect the immunoprecipitated complexes. Collected immunocomplexes were washed with 500 μL of respectively Low Salt Wash Buffer (150 mM NaCl, Tris-HCl pH 8 20 mM, EDTA 2 mM, Triton-X 1%, and SDS 0.1%), High Salt Wash Buffer (500 mM NaCl, Tris-HCl pH 8 20 mM, EDTA 2 mM, Triton-X 1%, and SDS 0.1%), LiCl Wash (0.25 M LiCl, Tris-HCl pH 8 10 mM, EDTA 1 mM, Igepal 1%, and Deoxycholate 1%), 1× TE (Tris-HCl pH 8 10 mM and EDTA). After washes, elution and cross-linking reversion were performed by heating at 65 °C overnight. DNA was recovered with Phenol/Chloroform and analyzed by 45 cycles of RT-PCR. BMP7 ChIP primers as described before (57). c-Myc ChIP primer: 5′-GGGGACTCAGTCTGGGTGG-3′ and 5′-AGCAACGCATTGCCACGTAT-3′.

### ChIP-Seq Library Preparation and Analysis.

For ChIP-Seq, the extracted DNA was ligated to specific adaptors, followed by deep sequencing on Illumina’s HiSeq 2000 according to the manufacturer’s instructions. ChIP-Seq for GPS2 was performed on two separate replicate experiments. Raw ChIP-Seq data are available at Gene Expression Omnibus (GEO) with the identifier GSE250040.

Quality control of the ChIP-Seq reads was performed using FastQC in default settings. ChIP-Seq read alignment as performed using Bowtie2 v2.2.7 (79) on human genome sequence (assembly hg38) with options *--local* and alignment files sorted and converted to bam using samtools v1.3 (80). Peak calling was performed using the function *findPeaks* of HOMER v4.11 with an option *style* parameter equal to histone (81). A reference cistrome for GPS2 and HDAC3 proteins for the wild-type condition was defined using the function *mergePeaks* of HOMER with parameter -d equal to *given*. Peaks were obtained on chromosome Y, and noncanonical chromosomes were removed. For each peak, the enrichment of GPS2 immuno-precipitation with respect to the input experiments was computed using the function *getDifferentialPeaks* of HOMER. The same function was used to compute the differential change of ChIP enrichment between the GPS2 KO experiments and those obtained in the WT condition. A peak was defined as differential enriched if associated with a *P*-value lower than 0.05. Between the two biological replicates of the GPS2 ChIP-Seq, the signal log2FC was averaged. At the same time, the *P*-values were combined using Fisher’s method implemented in the *sumlog* function of the *metap* R package.

The intensity of the ChIP-Seq signal was computed using Seqminer v1.3.4, considering a region of ±5 bp centered on the peak centers and divided into 50 bp bins (82). The number of reads mapped in each experiment was counted and subtracted by the number of reads obtained in the associated input sample for each bin. Peak genomic classification was performed using the *ChIPSeeker* R package (83) using the annotation from TxDb as a reference transcriptome. (*Hsapiens.UCSC.hg38.knownGene* R library). A region of −2 Kbp and +500 bp concerning gene TSS was considered the gene putative promoter. The functional enrichment analysis of ChIP-Seq peak-associated genes was performed using Enrichr (version of March 13th, 2022) (84). The analysis was performed separately on genes associated with peaks closer than 10 Kbp (proximal) and those associated with peaks far from this distance (distal). The ChIP-Seq peaks were also overlapped with chromatin states obtained by Ferrero et al. by integrating multiple ChIP-Seq experiments performed in MCF-7 maintained in a complete medium (46).

### RNA-Seq Library Preparation and Analysis.

Total RNA was harvested from cultured cells using the Qiagen RNeasy Plus Mini Kit according to the manufacturer’s instructions. Triplicates of each condition were sent to BU’s Microarray and Sequencing core for library preparation using Kapa RNA HyperPrep with RiboErase and run on two 75-paired end sequencing runs on Illumina NextSeq.

RNA-Seq data were analyzed with the STAR-RSEM analysis pipeline implemented in Docker4Seq (85). Specifically, raw reads were quality-controlled with FastQC and aligned on the Gencode v32 annotations using STAR (86). Then, gene expression quantification was performed using RSEM (87). Gene expression data were preprocessed using tximport (88), and the differential expression analysis was performed with DESeq2 (89) in default settings. A gene was differentially expressed if associated with an adjusted *P*-value < 0.001 and a median level in Transcript Per Million greater than 1 in at least one experimental condition.

### Statistical Analysis.

All the histogram data are the results of at least three independent experiments and are represented as the mean ± SEM. ChIP data are representative of two independent biological replicates. The differences between groups were compared using Student’s *t* test. Western blots are representative of three independent experiments.

## Supplementary Material

Dataset S01 (XLSX)

Dataset S02 (XLSX)

## Data Availability

Raw ChIP-Seq data are available at GEO with the identifier GSE250040 (45). The raw RNA-Seq reads are available in GEO with the identifier GSE250075 (48).
